# Biomechanical comparison of single-bundle versus double-bundle anterior cruciate ligament reconstruction: a meta-analysis

**DOI:** 10.1186/s43019-020-00033-8

**Published:** 2020-03-12

**Authors:** Jin-Young Oh, Kun-Tae Kim, Young-Jin Park, Hee-Chan Won, Jun-Il Yoo, Dong-Kyu Moon, Sung-Hee Cho, Sun-Chul Hwang

**Affiliations:** Department of Orthopaedic Surgery, Gyeongsang National University School of Medicine and Gyeongsang National University Hospital, 15, Jinju-daero 816 beon-gil, Jinju-si, Gyeongsangnam-do Republic of Korea 660-751

**Keywords:** Anterior cruciate ligament, Reconstruction, Single-bundle, Double-bundle, Biomechanical

## Abstract

**Background:**

Of the many issues regarding surgical techniques related to anterior cruciate ligament reconstruction (ACLR), single-bundle (SB) or double-bundle (DB) ACLR is one of the most debated topics. However, it is unclear which of the techniques yields better outcomes after ACLR for ACL injury. The purpose of this meta-analysis was to compare the benefits of SB versus DB ACLR in terms of biomechanical outcomes.

**Methods:**

The electronic databases MEDLINE, Embase, the Cochrane Central Register of Controlled Trials, Web of Science, and Scopus were searched for relevant articles comparing the outcomes of SB-ACLR versus DB-ACLR that were published until November 2019.

**Results:**

Seventeen biomechanical studies were included. The anterior laxity measured using the anterior drawer test showed significantly better results in DB-ACLR when compared with SB-ACLR. In addition, outcomes of the anterior tibial translation test under a simulated pivot shift presented with better results at low flexion and 30° in DB-ACLR, compared with SB-ACLR. However, there were no significant biomechanical differences between the groups in internal rotation.

**Conclusions:**

The present study demonstrated that both techniques for ACLR are associated with restoration of normal knee kinematics. DB-ACLR is superior to SB-ACLR in terms of restoration of anteroposterior stability. However, which technique yields better improvement in internal rotation laxity, and internal rotation laxity under a simulated pivot shift at a specific angle, remains unclear.

**Level of evidence:**

This is a level II meta-analysis.

## Background

There are various surgical methods for treating anterior cruciate ligament (ACL) injuries. Concerning these methods, there are differences of opinion among surgeons regarding single-bundle ACL reconstruction (SB-ACLR) and double-bundle ACL reconstruction (DB-ACLR). In past years, to reconstruct the injured ACL, the single-bundle (SB) procedure has been a standard surgical option. However, degenerative changes or arthrofibrosis remains a major concern after ACL reconstruction or other such surgical interventions [1, 2]. Arthrofibrosis or degenerative joint disease (DJD), which causes osteoarthritis, has been attributed to inefficient control of tibial rotation after SB-ACLR [3–7]. In addition, recent biomechanical studies have reported that SB-ACLR cannot restore normal anterior translation or rotatory laxity [5, 8]. To further improve the current SB-ACLR techniques and provide a greater understanding of the ACL anatomy, DB-ACLR techniques are being advocated to more closely reproduce the native anatomy of the ACL and potentially enhance the stability of the knee joint [9–11]. Despite an apparent theoretical advantage associated with the reconstruction of both bundles of the ACL, a consensus regarding the superiority of DB-ACLR over the conventional SB-ACLR has yet to be established [11–13]. Over the past decade, the biomechanics of ACL have been investigated in various experimental settings. Nonetheless, concerns regarding the choice of techniques for restoration of normal knee biomechanics persist. Among the various surgical issues of ACLR such as graft type, the methods of fixation, and the number of bundles, one of the critical controversies in ACLR relates to the role of DB reconstruction in biomechanical outcomes compared with SB reconstruction.

Recently, some studies [14–18] have shown a comparison of excellence between SB-ACLR or DB-ACLR, but the results are still unclear, and quantitative analyses of biomechanical results are insufficient. Thus, the purpose of the present study was to perform a meta-analysis to compare the biomechanical outcomes of SB- ACLR versus DB-ACLR and determine their relative effectiveness in anterior tibial translation (ATT), internal rotation, or pivot in the setting of ACLR. We hypothesized that DB-ACLR is better than SB-ACLR in controlling anterior stability and rotational stability.

## Materials and methods

### Study selection

We searched multiple databases to identify studies comparing biomechanical outcomes of ACLR in subjects who underwent SB or DB reconstruction. This study was based on the Cochrane Review methods, and the reporting was in accordance with the statement on Preferred Reporting Items for Systematic Reviews and Meta-Analyses (PRISMA). To identify relevant studies, we used the controlled vocabulary and free text words described in Additional file 2 to search the MEDLINE, Embase, Cochrane Central Register of Controlled Trials, Web of Science, and Scopus databases. We identified all relevant studies regardless of language, publication type (article, poster, conference article, instructional course lecture, etc.), publication journal, and publication year. This search was updated in November 2019 and includes reference lists of the studies and any review articles identified.

### Eligibility criteria

Studies were included in our investigation if (1) the studies compared biomechanical outcomes of SB-ACLR and DB-ACLR, (2) the subjects were human or human cadavers who received ACLR using SB or DB reconstruction, and (3) the comparative studies were controlled under laboratory settings. However, non-comparative studies of the effects of surgical technique, single-arm studies, which only described SB-ACLR or DB-ACLR, or studies that just recommended surgical treatment for ACL injury were excluded.

### Data collection and analysis

Two authors independently assessed the titles or abstracts of studies identified by the search strategy, and subsequently full papers were assessed for final inclusion. Uncertainty about study inclusion was resolved through discussion and consensus. Eligible data were independently abstracted onto predefined forms by the authors and reviewed for accuracy. We collected data on study characteristics (including authors, journal, publication year, study design, and level of evidence) and patient demographics (sex, age, number of subjects, graft type or surgical techniques [SB or DB] used for reconstruction) (Table 1). Results of biomechanical studies, including ATT, internal rotation, and ATT with pivot of the tibia and internal rotation, with standard deviation (SD) of demographic data and biomechanical outcomes between the two groups were determined.

**Table 1:** Summary of study characteristics

*Study*	*Journal*	*Year*	*Study design*	*Level of evidence*	*Bundle type*	*Age (years)*	*Sex (M:F)*	*Graft*
Albuquerque et al.	Clinics (Sao Paulo)	2007	CLS	Level of evidence (II)	SB:10	46.7 (27–67)	7:3	QBTG (10)
					DB:10			QBTG (10)
Sbihi et al.	Rev Chir Orthop Reparatrice Appar Mot	2004	CLS	Level of evidence (II)	SB:8	NP	NP	SemiT and gracilis (8)
					DB:8			SemiT and gracilis (8)
Ho et al.	Arthroscopy	2009	CLS	Level of evidence (II)	SB:8	68.8 (51–83)	4:4	SemiT and gracilis (8)
					DB:8			SemiT and gracilis (8)
Mae et al.	Arthroscopy	2001	CLS	Level of evidence (II)	SB:7	75 (67–86)	NP	SemiT and gracilis (7)
					DB:7			SemiT and gracilis (7)
Seon et al.	Am J Sports Med.	2010	CLS	Level of evidence (II)	SB:10	NP (47–60)	6:4	SemiT and/or gracilis (10)
					DB:10			SemiT and/or gracilis (10)
Yagi et al.	Am J Sports Med.	2002	CLS	Level of evidence (II)	SB:10	NP (44–60)	NP	SemiT and/or gracilis (10)
					DB:10			SemiT and/or racilis (10)
Yamamoto et al.	Am J Sports Med.	2004	CLS	Level of evidence (II)	SB:10	48.6 (39–55)	NP	SemiT and/or gracilis (10)
					DB:10			SemiT and/or gracilis (10)
Nohmi et al.	Sports Med Arthrosc Rehabil Ther Technol.	2012	CLS	Level of evidence (II)	SB:8	70.6 (18–93)	NP	SemiT and/or gracilis (8)
					DB:8			SemiT and/or gracilis (8)
Kondo et al.	Am J Sports Med.	2010	CLS	Level of evidence (II)	SB:8	62.1 (31–72)	NP	SemiT and gracilis (8)
					DB:8			SemiT and gracilis (8)
Goldsmith et al.	Am J Sports Med.	2013	CLS	Level of evidence (II)	SB:9	46.7 (46–58)	6:3	Bovine extensor tendon (9)
					DB:9			Bovine extensor tendon (9)
Lord et al.	Knee Surg Sports Traumatol Arthrosc	2016	CLS	Level of evidence (II)	SB:9	66 (57–78)	4:5	SemiT and gracilis (9)
					DB:9			SemiT and gracilis (9)
Gadikota et al.	Am J Sports Med.	2009	CLS	Level of evidence (II)	SB:8	NP (59–64)	NP	SemiT and gracilis (8)
					DB:8			SemiT and gracilis (8)
Kim et al.	Knee Surg Sports Traumatol Arthrosc	2015	CLS	Level of evidence (II)	SB:10	59.8 (49–69)	10:0	Quadriceps tendon (10)
					DB:10			Quadriceps tendon (10)
Herbort et al.	Am J Sports Med.	2015	CLS	Level of evidence (II)	SB:10	76.3 (63–86)	NP	Porcine flexor tendon (10)
					DB:10			Porcine flexor tendon (10)
Musahl et al.	Am J Sports Med.	2011	CLS	Level of evidence (II)	SB:10	62 (57–70)	NP	Gracilis (10)
					DB:10			Gracilis (10)
Komzák et al.	Eur J Trauma Emerg Surg	2017	CLS	Level of evidence (II)	SB:20	27.5 (17–42)	23:17	SemiT and/or gracilis (20)
					DB:20			SemiT and/or gracilis (20)
Suzuki et al.	Arthroscopy	2019	CLS	Level of evidence (II)	SB: 22	84 (72–92)	NP	SemiT (22)
					DB: 22			SemiT(22)s

*CLS* controlled laboratory study, *SB* single-bundle, *DB* double-bundle, *QBTG* quadriceps bone-tendon graft, *SemiT* semitendinosus tendon, *NP* not provided

### Assessment of methodological quality

Two investigators independently assessed the methodological qualities of each biomechanical study using the Quality Appraisal for Cadaveric Studies (QUACS) scale. The QUACS scale is highly reliable and presents strong construct validity for anatomical research [19]. Any disagreement between the authors was resolved through discussion or review by the third investigator.

### Statistical analysis

The main purpose of this review was to evaluate the restoration of normal knee kinematics after ACLR using SB and DB techniques. In biomechanical studies, restoration is based mainly on knee stability such as ATT, internal rotation, ATT under simulated pivot shift, and internal rotation of tibia under pivot shift. To evaluate reconstructed knee stability, we calculated the mean ± SD of each result of the SB and DB techniques and analyzed the differences in the outcome parameters between the groups. Review Manager (RevMan) version 5.3 (the Cochrane Collaboration, Software Update, Oxford) was used to estimate the overall pooled effect size for each outcome. A meta-analysis of the included studies was done using a random-effects model. For continuous outcomes, we conducted standard mean difference (SMD) calculation with a 95% confidence interval (CI) using the inverse variance method. Statistical heterogeneity among the studies was assessed using the *I*-squared (*I*^2^) index, with values of 25%, 50%, and 75% considered as low, moderate, and high, respectively, and Cochran’s Q statistic (chi-squared test). A *P* value < 0.10 was defined as a significant degree of heterogeneity.

## Results

### Identification of studies

**Fig. 1:**
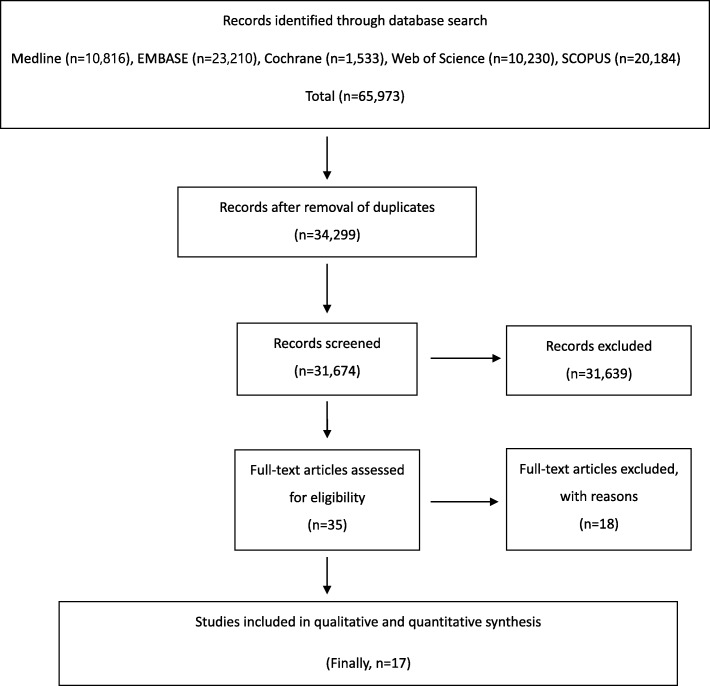
Preferred Reporting Items of Systematic Reviews and Meta-Analysis (PRISMA) flow diagram

A total of 65,973 relevant articles were initially identified. Of these, 31,674 were duplicated in the databases. After screening the remaining 34,299 articles using titles and abstracts, all but 35 were excluded because they were not relevant to the purpose of the present study. A full-text review of the 35 articles resulted in exclusion of articles because they lacked vital data, such as experimental outcomes. The exclusion criteria for articles included no investigation of the effect of individual ACLR techniques, biomechanical study using fine element method, review article, study evaluated patients who underwent conservative treatment for ACL injuries, or research involved animal studies. Finally, 17 biomechanical studies [20–36] were included for data extraction and meta-analysis (see Fig. 1).

### Quality of the included studies

To evaluate the methodologic quality, we used the QUACS scale for biomechanical studies (maximum 13 points). All subjects of the biomechanical studies had QUACS scale scores ≥10 points (range, 10–13) points, indicative of low risk of bias of the included biomechanical studies.

### Outcomes of knee stability in biomechanical studies

#### Anterior tibial translation

**Fig. 2:**
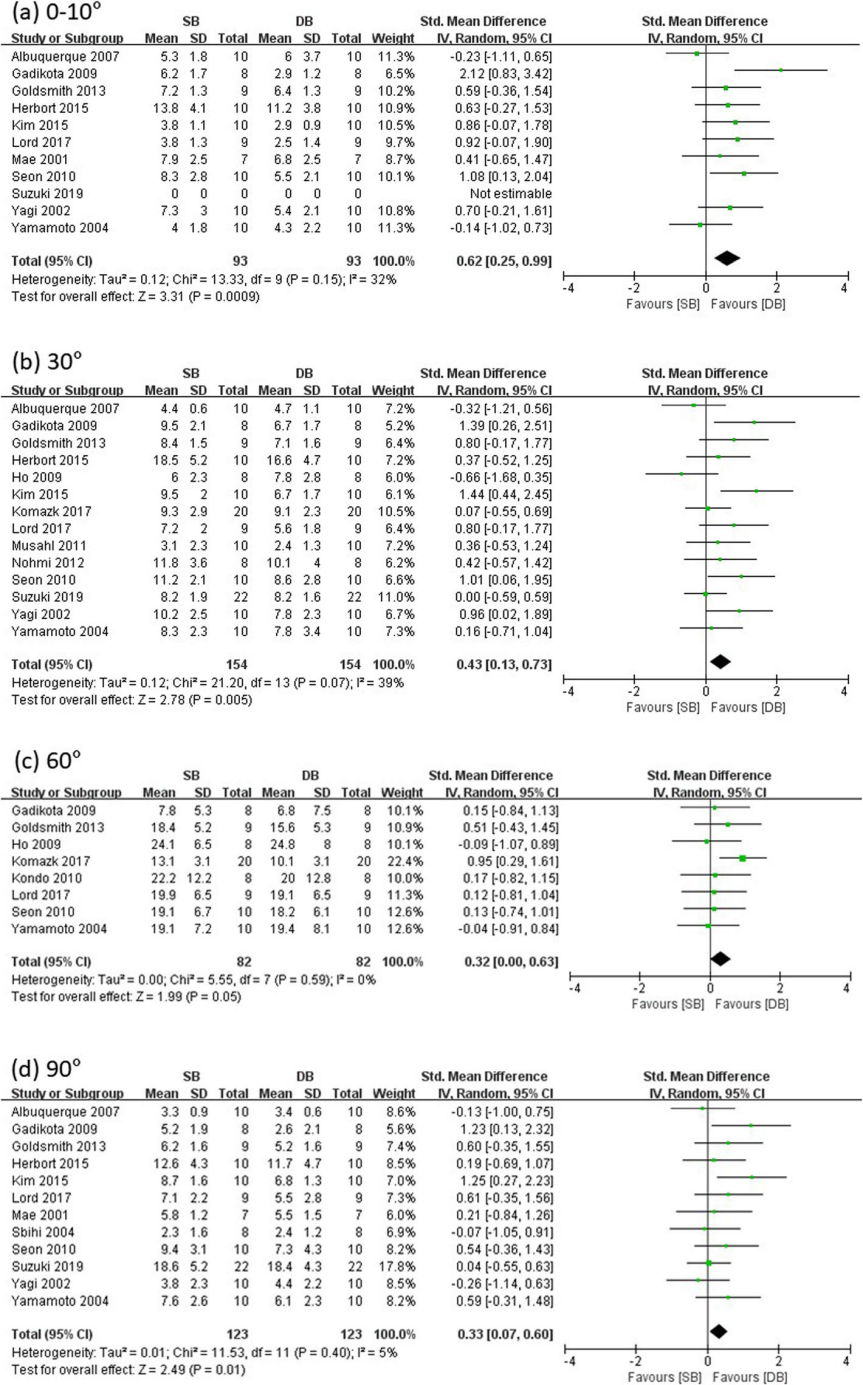
Forest plot showing standard mean difference in anterior tibial translation at different knee flexion angles: results of single-bundle versus double-bundle ACL reconstruction. **a** low flexion (0–10°), **b** 30°, **c** 60°, **d** 90°. *SB* single-bundle, *DB* double-bundle, *Std* standard, *SD* standard deviation, *IV* inverse variance, *CI* confidence interval *df* degrees of freedom

Fourteen studies reported ATT at low flexion (0–10°), 30°, 60°, and 90° knee flexion angle in SB and DB groups (SB group/DB group: 93/93 at low flexion, 112/112 at 30°, 109/109 at 60°, and 101/101 at 90°), and the remaining studies were excluded for insufficient data. Significant differences were found in ATT at all the knee flexion angles measured among the SB and DB groups (low flexion: SMD = 0.62, 95% CI = 0.25 to 0.99, *I*^2^ = 32%; 30°: SMD = 0.43, 95% CI = 0.13 to 0.73, *I*^2^ = 39%; 60°: SMD = 0.32, 95% CI = 0.00 to 0.63, *I*^2^ = 0%; 90°: SMD = 0.33, 95% CI = 0.07 to 0.60, *I*^2^ = 5%) (see Fig. 2). Therefore, anterior laxity measured using the anterior drawer test showed statistically significant results of more resistance in DB-ACLR when compared with SB-ACLR.

#### Internal rotation laxity

**Fig. 3:**
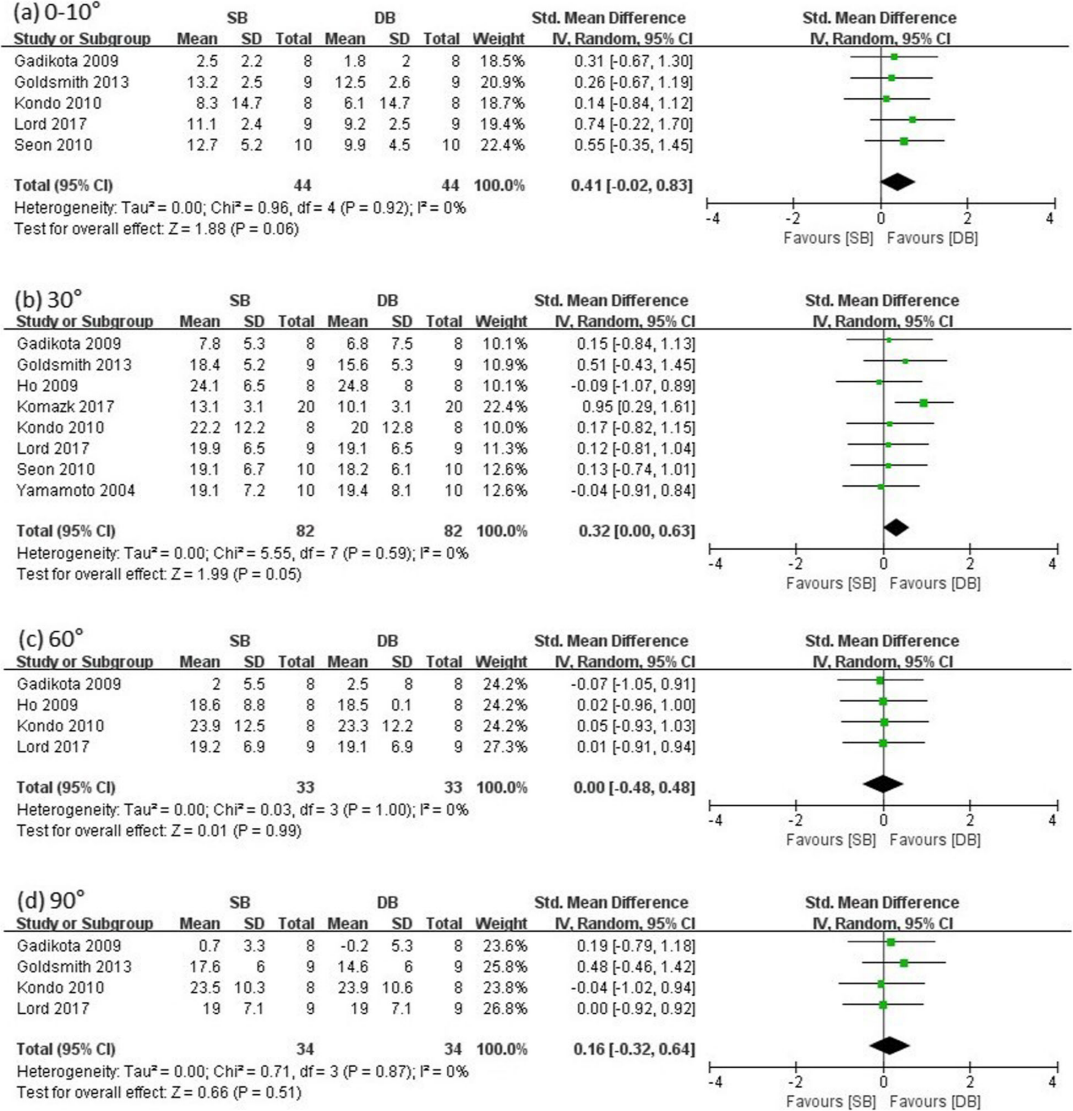
Forest plot showing standard mean difference in internal rotation at different knee flexion angles: results of single-bundle versus double-bundle ACL reconstruction. **a** low flexion (0–10°), **b** 30°, **c** 60°, **d** 90°. *SB* single-bundle, *DB* double-bundle, *Std* standard, *SD* standard deviation, *IV* inverse variance, *CI* confidence interval *df* degrees of freedom

Eight studies reported internal rotation laxity at different knee flexion angles in SB and DB groups (SB group/DB group: 44/44 at low flexion, 62/62 at 30°, 33/33 at 60°, 34/34 at 90°). The other studies did not include an internal rotation test. There were no significant differences in internal rotation laxity between the SB and DB groups at all evaluated knee flexion angles except 30° (low flexion: SMD = 0.41, 95% CI = − 0.02 to 0.83, *I*^2^ = 0%; 30°: SMD = 0.32, 95% CI = 0.00 to 0.63, *I*^2^ = 0%; 60°: SMD = 0.00, 95% CI = − 0.48 to 0.48, *I*^2^ = 0%; 90°: SMD = 0.16, 95% CI = − 0.32 to 0.64, *I*^2^ = 0%) (see Fig. 3). Therefore, internal rotation laxity showed statistically no difference between the two groups, and DB-ACLR was more resistant at 30° for the internal rotation test.

#### Anterior tibial translation under simulated pivot shift

**Fig. 4:**
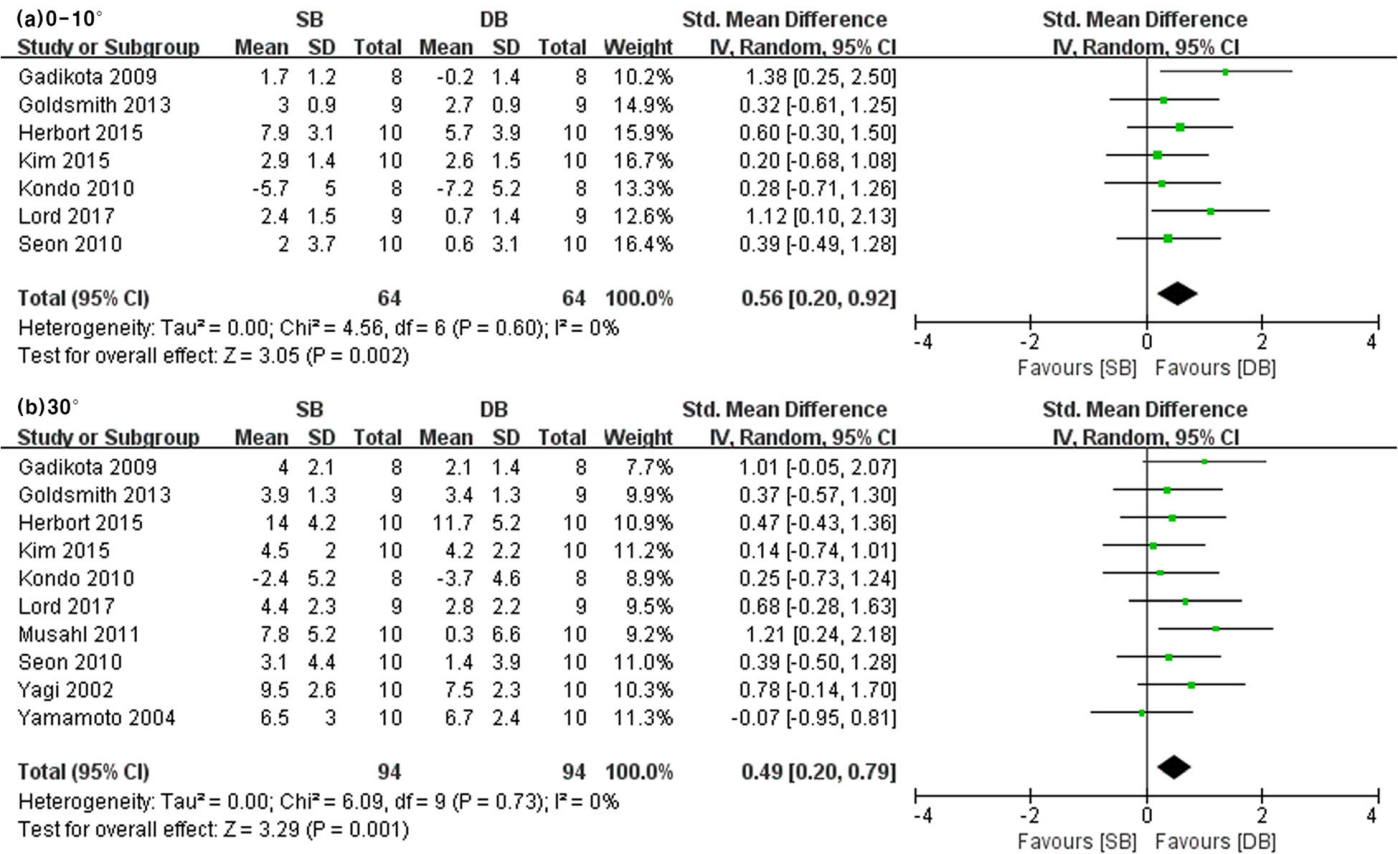
Forest plot showing standard mean difference in anterior tibial translation with pivot shift test at different knee flexion angles: results of single-bundle versus double-bundle ACL reconstruction. **a** low flexion (0–10°), **b** 30°. *SB* single-bundle, *DB* double-bundle, *Std* standard, *SD* standard deviation, *IV* inverse variance, *CI* confidence interval *df* degrees of freedom

Ten studies reported on ATT under simulated pivot shift at low and 30° knee flexion angles in SB and DB groups (SB group/DB group: 64/64 at low flexion, 94/94 at 30°). The other studies did not include information pertaining to ATT under simulated pivot shift. Significant differences were found in ATT under simulated pivot shift at low and 30° knee flexion angles between the SB and DB groups (low flexion: SMD = 0.56, 95% CI = 0.20 to 0.92, *I*^2^ = 0%; 30°: SMD = 0.49, 95% CI = 0.20 to 0.79, *I*^2^ = 0%) (see Fig. 4). Therefore, anterior rotational laxity measured using the anterior drawer test under a pivot shift test showed statistically significant results of more resistance in DB-ACLR when compared with SB-ACLR.

#### Internal rotation laxity under simulated pivot shift

**Fig. 5:**
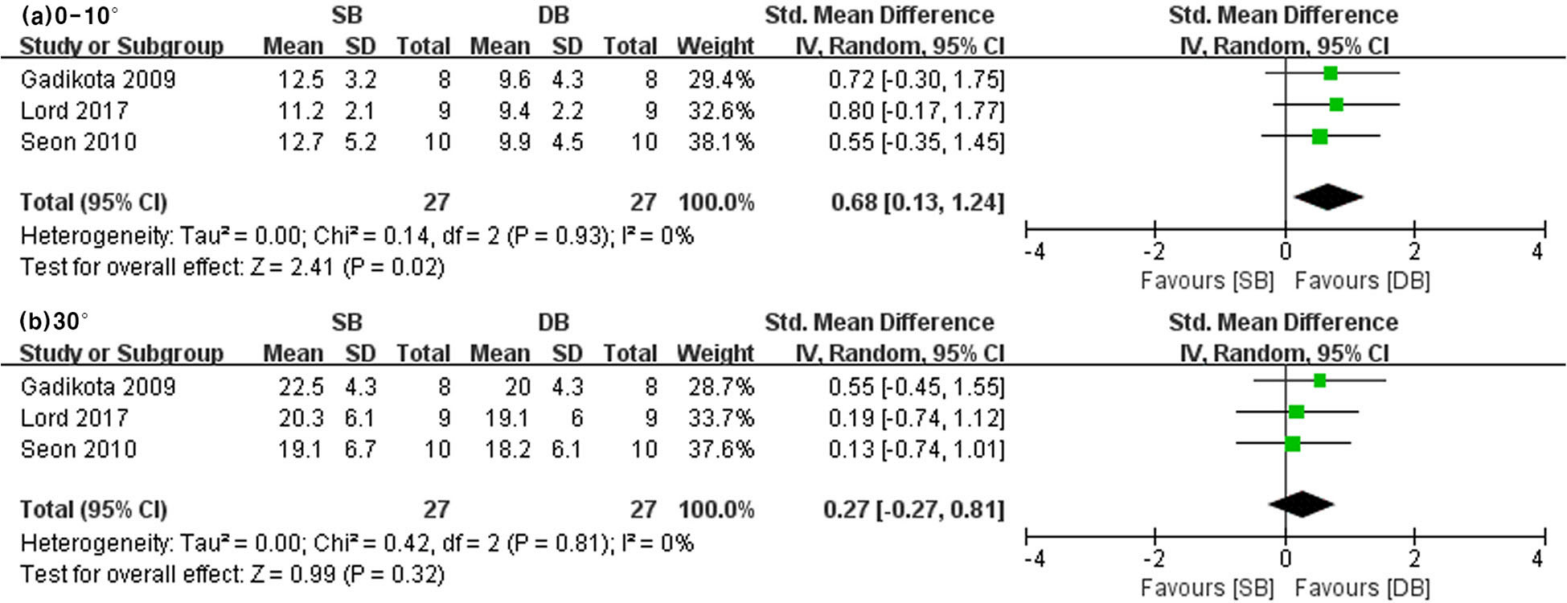
Forest plot showing standard mean difference in internal rotation with pivot shift test at different knee flexion angles: results of single-bundle versus double-bundle ACL reconstruction. **a** low flexion (0–10°), **b** 30°. *SB* single-bundle, *DB* double-bundle, *Std* standard, *SD* standard deviation, *IV* inverse variance, *CI* confidence interval *df* degrees of freedom

Three studies reported internal rotation of the tibia under pivot shift at low flexion and 30° knee flexion angles in SB and DB groups (SB group/DB group: 27/27 at low flexion, 27/27 at 30°). The other studies did not investigate internal rotation laxity under simulated pivot shift. There were differences in that DB-ACLR was more resistant in internal rotation laxity under simulated pivot shift at low knee flexion angles between the SB and DB groups. However, no significant differences were noted at 30° knee flexion angles between the SB and DB groups (low flexion: SMD = 0.68, 95% CI = 0.13 to 1.24, *I*^2^ = 0%; 30°: SMD = 0.27, 95% CI = − 0.27 to 0.81, *I*^2^ = 0%) (see Fig. 5).

## Discussion

In this meta-analysis, we assessed the evidence from controlled laboratory studies that compared outcomes with SB-ACLR versus DB-ACLR. The most important finding of this study was that ATT measured with the anterior drawer test favored DB-ACLR at all knee flexion angles. Another important finding showed that ATT measured under pivot shift at low and 30° knee flexion angles and internal rotation laxity under pivot shift at low flexion presented with superior outcome in DB-ACLR. However, we found no evidence of differences in internal rotation laxity associated with SB-ACLR and DB-ACLR at all knee flexion angles evaluated. Thus, the results of the meta-analysis supported our hypothesis that DB-ACLR is more effective than SB-ACLR in controlling anterior stability, although the association between the number of bundles and rotational instability remains unclear. Because of the low heterogeneity of results between the included studies, the meta-analysis strongly suggested that DB-ACLR was superior to SB-ACLR in terms of anterior laxity; however, the superiority of DB-ACLR for rotational laxity was inconclusive.

The ACL has a parallel array of collagen fascicles that are usually divided into anteromedial (AM) and posterolateral (PL) segments according to their attachment sites. The AM bundle plays a critical role in resisting tibial anterior drawer—the primary function of the ACL—while the PL bundle is tight near knee extension in tibial rotational laxity. The PL bundle was dominant near knee extension in a few studies [37–39], particularly when resisting anterior drawer, and its contribution reduced rapidly with knee flexion through 30° [40]. One of the main rationales for the introduction of DB-ACLR was to address the persistent anteroposterior (AP) instability and rotational instability of the knee after conventional surgical interventions [41]. Such rationales coincide with this study, which may represent evidence supporting DB-ACLR.

Several biomechanical studies have investigated the advantage in AP stability of DB-ACLR relative to SB-ACLR. In this meta-analysis performed on the basis of biomechanical studies, the DB-ACLR presented with more favorable stability at all angles with respect to AP stability. Despite controversies on whether DB-ACLR more closely restores AP laxity compared to SB-ACLR, many studies showed that the anterior laxity of both reconstructions did not differ statistically as compared to those of normal knee anterior stability. Some authors reported that DB-ACLR has no advantage in more anatomic structure and prevents ATT [22], and some reported that there was no clinically significant difference in AP stability between SB- and DB-ACLR [42, 43]. By contrast, others reported that DB-ACLR is superior biomechanically at all angles [33], and a meta-analysis [44, 45] reported that DB-ACLR is clinically more excellent. This is in line with the results of this study, and it is the result of strengthening the theoretical evidence of DB-ACLR.

The results of this study showed that DB-ACLR had a significant benefit in ATT at low flexion angle and 30° in the pivot shift test. In accordance with our results, Musahl et al. [36] reported that DB-ACLR had a more favorable outcome in terms of ATT in a pivot shift test at 30° when compared with SB-ACLR. Furthermore, Lord et al. [32] reported a lack of significant difference at 30°; however, DB-ACLR presented with a more favorable outcome in terms of ATT at low flexion, warranting further studies. Conversely, Herbort et al. [35] and Kondo et al. [30] reported no significant difference between the SB- and DB-ACLR groups at all angles in the pivot shift test. Goldsmith et al. [31] and Kim et al. [34] also showed that there was no significant difference between the two groups when ATT was measured in the pivot shift test at a low degree of flexion or 30°. In terms of ATT with the pivot shift test, our results indicate that DB-ACLR resulted in AP stability compared with SB-ACLR at low knee flexion angle and 30° in a pivot shift test between two groups. Gadikota et al. [33], Lord et al. [32], and Seon et al. [26] reported that internal rotation did not differ between the two groups at low flexion or 30° in the pivot shift test. However, such differences can be changed by experimental devices and conditions. A study using an accelerometer (Kisler, Switzerland) reported that the rotational acceleration was decreased by DB-ACLR to the intact level of the acceleration and that anatomic DB-ACLR is more dominant in restoring dynamic rotational laxity and biomechanically superior in terms of anatomic structure to SB-ACLR. Regarding internal rotation, DB-ACLR is more resistant in internal rotation at 30°, but Maeyama et al. [46] show no statistical difference between two groups in their study. Thus, a meta-analysis shows that DB-ACLR is more resistant at 30° internal rotation, but this may be due to the large number of knees studied.

The included biomechanical studies compared outcomes of AP stability, ATT with pivot shift, internal rotation laxity, and internal rotation with pivot shift of SB and DB techniques for ACL injuries. All the biomechanical studies scored ≥ 10 points on the QUACS scale, indicating a low risk of bias of the included studies and their eligibility for the meta-analysis. In addition, screening and data extraction were carried out by two independent and blinded reviewers, which is one of the strengths of our study. Although a previous meta-analysis focused on biomechanical studies of SB- and DB-ACLR, only 7 studies were involved in that analysis, which reported no significant differences in terms of biomechanical parameters. Thus, this study provides strong evidence regarding ACLR and valuable evidence in support of DB-ACLR.

However, despite its strengths, our study has some limitations. First of all, this study is a meta-analysis based on time zero studies that were not reflected after a period of time in vivo such as healing and biological responses. While it is not possible to conclude how the results may influence patient outcomes and clinical practice other than the outcome on the day of surgery, time zero studies are commonly suitable for providing guidelines for clinical practice in terms of procedure or device selection, as they provide baseline information. Second, a relatively small number of studies were included in this meta-analysis. In addition, several included studies used elderly cadaveric knees for ACLR; hence, the tissue properties and bone density may have differed from those of younger patients with sports injuries, which may carry a risk of bias. Moreover, there are few previously published original articles on this topic, which is an absolute limitation. The inclusion of 17 in vitro studies and the disparities of testing protocols may significantly contribute to the generation of bias when comparing mean differences of these studies. In order to compensate for such bias, additional studies were included, and powerful evidence was drawn through calculation of the SMD, as compared with previous meta-analyses. Third, technical factors of surgery that may affect results following ACLR need to be controlled, including those associated with SB or DB techniques, as well as graft characteristics, tensioning protocol, and graft fixation methods. In other words, the methodologies of studies included in this meta-analysis showed heterogeneity.

Meta-analysis is a process to arrive at powerful conclusions by analyzing studies on a specific topic. However, it is impossible to include studies exclusively with a similar methodology, and the inclusion criteria suggested in the article were strictly adopted to minimize bias when selecting the studies and were based on recommendations from Cochrane Collaboration guidelines. Therefore, the risk of bias was minimized by including only comparative studies conducted under the same conditions, using a random-effects model, and calculating the SMD statistically. In the future, prospective studies that control for such independent factors through high-quality medical research are needed.

## Conclusions

In conclusion, this meta-analysis suggested that DB-ACLR is biomechanically superior to SB-ACLR in terms of restoration of anterior laxity of the knee. However, the results on which technique provides better restoration in rotational instability remain inconclusive. To verify and strengthen our results, more high-quality randomized controlled trials are required.

## Supplementary information

**Additional file 1.** Supplemental materials I. Anterior tibial translation with anterior drawer force after single-bundle anterior cruciate ligament reconstruction and double-bundle anterior cruciate ligament reconstruction at the different knee flexion angles.

**Additional file 2.** Electronic Search Strategy on Each Database.

## Data Availability

Not applicable.

## Abbreviations

ACL: Anterior cruciate ligament
AM: Anteromedial
AP: Anteroposterior
ATT: Anterior tibial translation
CI: Confidence interval
DB-ACLR: Double-bundle anterior cruciate ligament reconstruction
ITB: Iliotibial band
PL: Posterolateral
PRISMA: Preferred Reporting Items for Systematic Reviews and Meta-Analyses
QUACS: Quality Appraisal for Cadaveric Studies
SB: Single-bundle
SB-ACLR: Single-bundle anterior cruciate ligament reconstruction
SD: Standard deviation
SMD: Standard mean difference
