# Supplementary material for: Biomechanical comparison of single-bundle versus double-bundle anterior cruciate ligament reconstruction: a meta-analysis
Source: Knee Surg Relat Res. 2020 Mar 12;32:14. doi: 10.1186/s43019-020-00033-8 (PMC7219200; doi:10.1186/s43019-020-00033-8)
Supplement: Supplementary file 1 — Additional file 1. Supplemental materials I. Anterior tibial translation with anterior drawer force after single-bundle anterior cruciate ligament reconstruction and double-bundle anterior cruciate ligament reconstruction at the different knee flexion angles. [file 43019_2020_33_MOESM1_ESM.docx]

Supplemental materials I. Anterior tibial translation with anterior drawer force after single-bundle anterior cruciate ligament reconstruction and double-bundle anterior cruciate ligament reconstruction at the different knee flexion angles.

|  | *Reconstruction techniques* | *Anterior tibial translation at different knee flexion angles (mm)* | | | |
| --- | --- | --- | --- | --- | --- |
|  |  | *Low flexion (0°-10°)* | *30°* | *60°* | *90°* |
| Albuquerque et al. | SB | 5.3±1.8 | 4.4±0.6 | 3.8±0.6 | 3.3±0.9 |
|  | DB | 6.0±3.7 | 4.7±1.1 | 3.7±0.7 | 3.4±0.6 |
| Ho et al. | SB | NP | 6.0±2.3 | 6.0±3.7 | NP |
|  | DB | NP | 7.8±2.8 | 5.2±2.9 | NP |
| Mae et al. | SB | 7.9±2.5 | NP | 7.7±1.9 | 5.8±1.2 |
|  | DB | 6.8±2.5 | NP | 7.3±1.7 | 5.5±1.5 |
| Sbihi et al. | SB | NP | NP | 3.1±1.1 | 2.3±1.6 |
|  | DB | NP | NP | 2.6±1.5 | 2.4±1.2 |
| Seon et al. | SB | 8.3±2.8 | 11.2±2.1 | 10.1±2.6 | 9.4±3.1 |
|  | DB | 5.5±2.1 | 8.6±2.8 | 8.0±3.7 | 7.3±4.3 |
| Yagi et al. | SB | 7.3±3.0 | 10.2±2.5 | 6.9±2.0 | 3.8±2.3 |
|  | DB | 5.4±2.1 | 7.8±2.3 | 6.1±2.2 | 4.4±2.2 |
| Yamamoto et al. | SB | 4.0±1.8 | 8.3±2.3 | 8.9±2.1 | 7.6±2.6 |
|  | DB | 4.3±2.2 | 7.8±3.4 | 7.6±2.4 | 6.1±2.3 |
| Nohmi et al. | SB | NP | 11.8±3.6 | NP | NP |
|  | DB | NP | 10.1±4.0 | NP | NP |
| Herbort et al. | SB | 13.8±4.1 | 18.5±5.2 | 15.8±5.0 | 12.6±4.3 |
|  | DB | 11.2±3.8 | 16.6±4.7 | 14.5±4.7 | 11.7±4.7 |
| Musahl et al. | SB | NP | 3.1±2.3 | NP | NP |
|  | DB | NP | 2.4±1.3 | NP | NP |
| Kim et al. | SB | 3.8±1.1 | 9.5±2.0 | 7.8±2.4 | 8.7±1.6 |
|  | DB | 2.9±0.9 | 7.8±2.0 | 6.2±1.8 | 6.8±1.3 |
| Gadikota et al | SB | 6.2±1.7 | 9.5±2.1 | 7.4±1.9 | 5.2±1.9 |
|  | DB | 2.9±1.2 | 6.7±1.7 | 2.9±2.1 | 2.6±2.1 |
| Goldsmith et al. | SB | 7.2±1.3 | 8.4±1.5 | 8.3±1.8 | 6.2±1.6 |
|  | DB | 6.4±1.3 | 7.1±1.6 | 6.8±1.5 | 5.2±1.6 |
| Lord et al. | SB | 3.8±1.3 | 7.2±2.0 | 7.3±1.7 | 7.1±2.2 |
|  | DB | 2.5±1.4 | 5.6±1.8 | 5.8±1.9 | 5.5±2.8 |
| Komzák et al. | SB | NP | 9.3±2.9 | NP | NP |
|  | DB | NP | 9.1±2.3 | NP | NP |
| Suzuki et al. | SB | 5.5±2.0 | 8.2±1.9 | 13.7±4.0 | 18.6±5.2 |
|  | DB | 4.5±1.6 | 8.2±1.6 | 13.2±3.6 | 18.4±4.3 |

SB, single-bundle; DB, double-bundle; NP, not provided

Supplemental materials II. Internal rotation in response to internal rotation torques after single-bundle and double-bundle anterior cruciate ligament reconstruction.

|  | *Reconstruction techniques* | *Internal rotation at different knee flexion angles (deg)* | | | |
| --- | --- | --- | --- | --- | --- |
|  |  | *Low flexion (0°-10°)* | *30°* | *60°* | *90°* |
| Ho et al. | SB | NP | 24.1±6.5 | 18.6±8.8 | NP |
|  | DB | NP | 24.8±8.0 | 18.5±0.0 | NP |
| Seon et al. | SB | 12.7±5.2 | 19.1±6.7 | NP | NP |
|  | DB | 9.9±4.5 | 18.2±6.1 | NP | NP |
| Yamamoto et al. | SB | NP | 19.1±7.2 | NP | NP |
|  | DB | NP | 19.4±8.1 | NP | NP |
| Gadikota et al. | SB | 2.5±2.2 | 7.8±5.3 | 2.0±5.5 | 0.7±3.3 |
|  | DB | 1.8±2.0 | 6.8±7.5 | 2.5±8.0 | -0.2±5.3 |
| Kondo et al. | SB | 8.3±14.7 | 22.2±12.2 | 23.9±12.5 | 23.5±10.3 |
|  | DB | 6.1±14.7 | 20.0±12.8 | 23.3±12.2 | 23.9±10.6 |
| Goldsmith et al. | SB | 13.2±2.5 | 18.4±5.2 | NP | 17.6±6.0 |
|  | DB | 12.5±2.6 | 15.6±5.3 | NP | 14.6±6.0 |
| Lord et al. | SB | 11.1±2.4 | 19.9±6.5 | 19.2±6.9 | 19.0±7.1 |
|  | DB | 9.2±2.5 | 19.1±6.5 | 19.1±6.9 | 19.0±7.1 |
| Komzák et al. | SB | NP | 13.1±3.1 | NP | NP |
|  | DB | NP | 10.1±3.1 | NP | NP |

SB, single-bundle; DB, double-bundle; NP, not provided

Supplemental materials III. Anterior tibial translation under simulated pivot- shift (mean±SD) in single-bundle and double-bundle anterior cruciate ligament reconstruction.

|  | *Reconstruction techniques* | *Anterior tibial translation with pivot at different knee flexion angles (mm)* | | | |
| --- | --- | --- | --- | --- | --- |
|  |  | *Low flexion (0°-10°)* | *30°* | *60°* | *90°* |
| Seon et al. | SB | 2.0±3.7 | 3.1±4.4 | NP | NP |
|  | DB | 0.6±3.1 | 1.4±3.9 | NP | NP |
| Yagi et al. | SB | NP | 9.5±2.6 | NP | NP |
|  | DB | NP | 7.5±2.3 | NP | NP |
| Yamamoto et al. | SB | NP | 6.5±3.0 | NP | NP |
|  | DB | NP | 6.7±2.4 | NP | NP |
| Tsai et al. | SB | 5.0±0.8 | 9.1±1.7 | 8.0±2.5 | 6.6±2.0 |
|  | DB | 6.7±2.3 | 9.3±3.1 | 9.3±4.7 | 9.6±5.6 |
| Herbort et al. | SB | 7.9±3.1 | 14.0±4.2 | 13.3±4.8 | 12.9±4.1 |
|  | DB | 5.7±3.9 | 11.7±5.2 | 12.3±5.6 | 12.4±5.8 |
| Musahl et al. | SB | NP | 7.8±5.2 | NP | NP |
|  | DB | NP | 0.3±6.6 | NP | NP |
| Kim et al. | SB | 2.9±1.4 | 4.5±2.0 | NP | NP |
|  | DB | 2.6±1.5 | 4.2±2.2 | NP | NP |
| Gadikota et al. | SB | 1.7±1.2 | 4.0±2.1 | NP | NP |
|  | DB | -0.2±1.4 | 2.1±1.4 | NP | NP |
| Kondo et al. | SB | -5.7±5.0 | -2.4±5.2 | 2.0±4.8 | 8.7±5.9 |
|  | DB | -7.2±5.2 | -3.7±4.6 | 1.9±4.6 | 8.6±5.2 |
| Goldsmith et al. | SB | 3.0±0.9 | 3.9±1.3 | NP | NP |
|  | DB | 2.7±0.9 | 3.4±1.3 | NP | NP |
| Lord et al. | SB | 2.4±1.5 | 4.4±2.3 | NP | NP |
|  | DB | 0.7±1.4 | 2.8±2.2 | NP | NP |

SB, single-bundle; DB, double-bundle

Supplemental materials IV. Internal rotation of tibia under simulated pivot- shift (mean±SD) in single-bundle and double-bundle posterior cruciate ligament reconstruction.

|  | *Reconstruction techniques* | *Change in internal rotation (deg)* | | | |
| --- | --- | --- | --- | --- | --- |
|  |  | *Low flexion (0°-10°)* | *30°* | *60°* | *90°* |
| Gadikota et al | SB (8) | 12.50±3.2 | 22.50±4.3 | NP | NP |
|  | DB (8) | 9.64±4.3 | 20.00±4.3 | NP | NP |
| Seon et al. | SB (10) | 12.7±5.2 | 19.1±6.7 | NP | NP |
|  | DB (10) | 9.9±4.5 | 18.2±6.1 | NP | NP |
| Lord et al. | SB (9) | 11.2±2.1 | 20.3±6.1 | NP | NP |
|  | DB (9) | 20.3±2.2 | 19.1±6.0 | NP | NP |

SB, single-bundle; DB, double-bundle
