# Supplementary material for: Biomechanical comparison of single-bundle versus double-bundle anterior cruciate ligament reconstruction: a meta-analysis
Source: Knee Surg Relat Res. 2020 Mar 12;32:14. doi: 10.1186/s43019-020-00033-8 (PMC7219200; doi:10.1186/s43019-020-00033-8)
Supplement: Supplementary file 2 — Additional file 2. Electronic Search Strategy on Each Database. [file 43019_2020_33_MOESM2_ESM.docx]

Additional file 2

MEDLINE

1. “Anterior Cruciate Ligament”[tiab] OR “Anterior Cruciate Ligaments”[tiab] 17,838

2. "Anterior Cruciate Ligament"[Mesh] 10,606

3. 1 OR 2 19,992

4. "Reconstructive Surgical Procedures"[Mesh] 201,372

5. Reconstructive[tiab] OR reconstruction[tiab] OR transtibial[tiab] OR transportal [tiab] OR inside-out [tiab] OR outside-in [tiab] 221,994

6. Single-Bundle[tiab] OR Double-Bundle[tiab] OR ((Single[tiab] OR Double[tiab]) AND Bundle[tiab]) 5,799

7. 4 OR 5 OR 6 377,978

8. 3 AND 7 12,103

9. 8 NOT "review"[Publication Type] OR "review literature as topic"[MeSH Terms] 10,816

EMBASE

1. 'knee ligament'/de OR 'knee cruciate ligament'/exp 23,888

2. "Anterior Cruciate Ligament":ab,ti 20,450

3. 1 OR 2 33,713

4. 'knee ligament surgery'/de OR 'posterior cruciate ligament reconstruction'/exp OR 'ligament surgery'/exp 16,775

5. Reconstructive:ab,ti OR reconstruction:ab,ti OR transtibial:ab,ti OR inside-out:ab,ti OR outside-in:ab,ti 269,953

6. Single-Bundle:ab,ti OR Double-Bundle:ab,ti OR ((Single:ab,ti OR Double:ab,ti) AND Bundle:ab,ti) 7,401

7. 4 OR 5 OR 6 18,056,534

8. 3 AND 7 25,312

9. 8 NOT ('conference review'/it OR 'review'/it) 23,210

COCHRANE

1. “Anterior Cruciate Ligament” OR “Anterior Cruciate Ligaments”:ti,ab,kw 2,411

2. MeSH descriptor: [Posterior Cruciate Ligament] explode all trees 655

3. 1 OR 2 2,411

4. MeSH descriptor: [Reconstructive Surgical Procedures] explode all trees 8,034

5. Reconstructive OR reconstruction OR transtibial OR inside-out OR outside-in 11,264

6. Single-Bundle OR Double-Bundle OR ((Single OR Double) AND Bundle) 784

7. 4 OR 5 OR 6 18,084

8. 3 AND 7 1,868

9. 8/trials 1,533

Web Of Science (WOS)

1. TOPIC: (“Anterior Cruciate Ligament” OR “Anterior Cruciate Ligaments”) OR TITLE: (“Anterior Cruciate

Ligament” OR “Anterior Cruciate Ligaments”) 20,093

2. TOPIC: (Reconstructive OR reconstruction OR transtibial OR inside-out OR outside-in OR Single-Bundle

OR Double-Bundle OR ((Single OR Double) AND Bundle)) OR TITLE: (Reconstructive OR reconstruction OR

transtibial OR inside-out OR outside-in OR Single-Bundle OR Double-Bundle OR ((Single OR Double) AND

Bundle)) 429,589

3. 1 AND 2 12,403

4. 3 Refined by: [excluding] DOCUMENT TYPES: ( REVIEW ) 10,230

SCOPUS

1. TITLE-ABS ( "Anterior Cruciate Ligament" OR " Anterior Cruciate Ligaments" ) 21,158

2. INDEXTERMS (Anterior cruciate ligament) 22,649

3. 1 OR 2 2,777,149

4. TITLE-ABS ("Reconstructive Surgical Procedures") 280

5. Reconstructive OR reconstruction OR transtibial OR inlay OR Single-Bundle OR Double-Bundle OR ((Single OR Double) AND Bundle) 2,169,956

6. 4 OR 5 2,169,956

7. 3 AND 6 20,184

8. 7 AND EXCLUDE ( DOCTYPE , "re" ) 20,fa184
